# Determinants of COVID-19 vaccine preference: A survey study in Japan

**DOI:** 10.1016/j.ssmph.2021.100902

**Published:** 2021-08-24

**Authors:** Keisuke Kawata, Masaki Nakabayashi

**Affiliations:** Institute of Social Science, The University of Tokyo, Hongo 7-3-1, Bunkyo, 103-0033, Tokyo, Japan

**Keywords:** COVID-19 vaccines, Randomized conjoint experiment, Domestic development, Domestic clinical trial, Geopolitical concerns, Herd immunity free-riding

## Abstract

**Objective:**

Vaccination is a critical measure for containing the COVID-19 pandemic. We survey the determinants that affect the preference for COVID-19 vaccines in Japan, a vaccine hesitant nation.

**Setting and design:**

We conducted a randomized conjoint analysis survey of the preference for vaccines on the Internet by recruiting a nonprobability sample of 15,000 Japanese adults. The survey assigned 5 choice tasks to the respondents. In each task, the respondents evaluated 2 hypothetical COVID-19 vaccines and were asked which they would choose. The vaccine attributes included efficacy, major and minor adverse side effects, country of vaccine development and clinical trial, and vaccine type.

**Treatment:**

The choice task asked the participants to select a vaccine from 2 hypothetical vaccines as an optional vaccine or select a vaccine as mandated one with a probability of 0.5 for each.

**Results:**

Compared to China-developed vaccines, domestically developed or US-developed vaccines raised the choice probability by 37.3 and 27.4 percentage points, respectively. A domestic clinical trial increased the choice probability by 14.8, an increase in efficacy from 50% to 90% increased that by 18.0, and a decrease in the risk of severe adverse side effects from 1 per 10 thousand to 1 per 1 million increased that by 16.9 percentage points, respectively. The vaccine type was irrelevant. Making vaccination compulsory increased the choice probability of China- and Russia-developed vaccines by 0.6 and 0.4, high-risk vaccines by 0.5, and a modestly effective (70%) vaccine by 0.4 percentage points, respectively. General vaccination hesitancy, political positions, demographic characteristics, education, and income were irrelevant.

**Conclusions:**

A domestically developed vaccine with a domestic clinical trial could substantially increase the preference for the vaccine. Making vaccination compulsory could modestly reduce the penalty for a vaccine with adverse side effects, geopolitical, and efficacy concerns, possibly through mitigating free-riding concerns to achieve herd immunity.

## Highlights

• We implement a randomized conjoint experiment on preference of COVID-19 vaccines.
• We asked 15,000 Japanese adults which to prefer between two hypothetical vaccines.
• As a treatment, we randomly assigned voluntary and compulsory vaccination scenarios.
• Domestic development and clinical trial were substantially preferred.
• Compulsory vaccination modestly raised preference for riskier vaccines.

## 1 Introduction

Hesitancy regarding vaccines is a factor that hinders the achievement of herd immunity, and the reason and background characteristics of such hesitancy could be multidimensional (MacDonald (2015); Seanehia et al. (2017); Anderson et al. (2020); Keske et al. (2020); Niño et al. (2021); and Saied et al. (2021)). Such hesitancy is a critical issue amid the COVID-19 pandemic with possible adverse side effects of vaccines (Malik et al. (2020); Bell et al. (2020); Callaghan et al. (2021); Caserotti et al. (2021); Detoc et al. (2020); Ward et al. (2020); Fisk (2021); Karlsson et al. (2021, p. 110590); Latkin et al. (2021); Mercadante and Law (2020); Ruiz, Bell, and Feb (2021); Schoch-Spana et al. (2020); Troiano and Nardi (2021); Wong et al. (2021)) along with the pecuniary costs of vaccination (García and Cerda (2020)). If we believe that vaccination could be effective in containing the pandemic, then we must find a way to persuade people to get vaccinated. One possible approach is to identify people’s concerns regarding the entire process of development, clinical trials, and provisioning and address the issues as attempted by Gagneux-Brunon et al. (2021). Among the possible settings used to measure stated preferences, randomized conjoint experiments (Hainmueller et al. (2014) and Hainmueller et al. (2015)) in which vaccine attributes are randomly combined are effective in identifying the aspects people like and dislike. Such studies were implemented in China prior to the COVID-19 pandemic (Sun et al. (2020)), and, to achieve better vaccination against COVID-19, were conducted in the US (Kreps et al. (2020); Motta (2021); and Kaplan and Milstein (2021)) and France (Schwarzinger et al. (2021)). These studies report that efficacy has a positive impact while the adverse side effect risk and vaccine development in foreign countries, notably with geopolitical concerns, have a negative impact on hypothetical vaccine choice.

Japan is not an exception to vaccination hesitancy (de Figueiredo et al. (2020)). Furthermore, as of March 2021, when our survey was implemented, Japan had not yet completed domestic development of a vaccine against COVID-19. Thus, we implemented a randomized conjoint experiment involving 15,000 respondents to identify the factors associated with a more acceptable hypothetical vaccine. Additionally, an ultimate means to extend coverage of vaccination is to make it compulsory. Compulsory vaccination removes the possibility of free-riding in building herd immunity and hence might help, for instance, a risky vaccine prevail. Therefore, in our conjoint experiment, we randomly assigned one of optional and compulsory vaccination scenarios to the respondents and analyzed whether these scenarios resulted in different outcomes.

## 2 Methods

We conducted an online survey from February to March 2021 to measure individual valuations of hypothetical COVID-19 vaccines. The Ethical Review Board of the Institute of Social Science, The University of Tokyo approved this study. The survey format was provided by Rakuten Insight.^1^ Each question was assigned based on a previous question, and the respondents were not allowed to return to a previous question once they responded to or skipped the question. Each respondent was asked which of two hypothetical vaccines she/he would prefer.

### 2.1 Randomized conjoint analysis

Randomized conjoint analysis originates from market research and has recently been applied to public health, including research aiming to identify the factors that affect attitudes toward COVID-19 vaccination in the US (Kreps et al. (2020) and Motta (2021)) and in France Scharzinger et al. (2021).

### 2.2 Survey

#### 2.2.1 Aim

Our aim is to identify the type of vaccines that would be more likely to be accepted if people have to get vaccinated. The supply bottleneck of COVID-19 vaccines is currently an issue. However, eventually, the supply will meet the demand. Then, a critical issue becomes the vaccine hesitancy of the public. At this stage, the government must persuade the public to get vaccinated. The cost of the implementation of such a policy could be reduced if the authorities knew the types of vaccines that are less disliked. To address this issue explicitly, we do not allow the respondents to opt out of vaccination.

At a transitional stage until the supply of the most preferred vaccines meets the demand, the government might have to persuade the public to take less preferred substitutes. For this, the government may make vaccination compulsory. We also estimate the effects of mandating vaccination.

#### 2.2.2 Treatment: two scenarios

Before introducing the hypothetical vaccines, we randomly presented two different scenarios to the respondents. One scenario asked the respondents which vaccine she/he preferred for herself/himself. The other scenario asked the respondents which vaccine should be selected if the government made vaccination compulsory. A theoretically possible difference between these scenarios is that the former self-choice scenario might include free-rider problem effects. Not all individuals need to get vaccinated to achieve herd immunity. For example, if most people other than one individual get vaccinated, the unvaccinated person would be protected by the herd immunity built by the efforts of other people and might have incentive to avoid riskier vaccinations. The latter scenario of compulsory vaccination might reduce such free-riding concerns.

#### 2.2.3 Attributes

In this survey, the respondents were informed of the vaccines being supplied and under development. Each hypothetical vaccine profile consisted of the following 6 categories: a) risk of severe adverse side effects resulting in hospitalization or death, b) risk of mild adverse side effects resulting in flu-like symptoms, c) efficacy in protecting against severe symptoms, d) type of vaccine, namely, messenger RNA, inactivated virus, or weakened virus, e) country of development (Japan, US, China, UK, or Russia), and f) country of clinical trial (in Japan or not in Japan). Notably, our aim is to identify the respondents’ usual preference for vaccines given their knowledge; therefore, we did not provide additional information regarding the vaccine types.

The attributes are summarized in Table 1. For each category, one attribute level was randomly drawn to characterize a hypothetical vaccine. Two such hypothetical vaccines were shown to the respondents, who were then asked to make a choice. We did not allow the respondents to opt out; therefore, between two levels of an attribute, the relatively disliked level was selected with a probability less than 0.5, and the preferred level was selected with a probability greater than 0.5. Each respondent participated in 5 selection tasks, resulting in 10 outcomes, including the chosen vaccine and unchosen vaccine in each task. In summary, we collected 10 (outcomes) × 15,000 respondents = 150,000 samples.

**Table 1:** Attributes and attribute levels.

Country of development	Japan	Country of clinical trial	in Japan
	US		not in Japan
	China		
	UK		
	Russia		
Risk of severe side effects (hospitalization or death)	1 in 10,000	Mild side effects (flu-like symptoms)	1 in 10
	1 in 1,000,000		1 in 30
Efficacy (protection against severe symptoms)	50%	Type of vaccine	Messenger RNA
	70%		Inactivated virus
	90%		Weakened virus

### 2.3 Data collection

We recruited 15,000 Japanese adults using the Rakuten Insight platform between February 16 and March 15, 2021. The detailed information of the Rakuten Insight respondent pool is available on its website.^2^ In addition to their demographic characteristics, such as gender, age, income, and education, we asked about their past experience with COVID-19 infection, general attitude toward vaccination, past vaccination or vaccination refusal experience, trust in vaccine licensing, and trust in doctors’ advice because these factors might affect their preference for a vaccine. The descriptive statistics are presented in Table 2.

**Table 2:** Descriptive statistics of the background characteristics.

Statistic	N	Mean	St. Dev.	Min	Max
Gender	15,000	0.504	0.500	0	1
Age	15,000	47.610	13.901	18	79
Marital status	14,936	0.632	0.482	0.000	1.000
Number of children	14,975	1.112	1.126	0.000	5.000
Previous COVID-19 infection	14,973	0.022	0.148	0.000	1.000
Previous COVID-19 infection: NA	14,973	0.016	0.125	0.000	1.000
Experience with vaccinations other than COVID-19	14,906	0.470	0.499	0.000	1.000
Whether vaccination is considered safe	14,904	0.643	0.479	0.000	1.000
Whether vaccination is considered important	14,645	0.810	0.392	0.000	1.000
Whether trust in vaccine licensing by MHLW exists	14,886	0.693	0.461	0.000	1.000
Experience with refusing vaccination	14,923	0.187	0.390	0.000	1.000
Experience with postponing vaccination	14,908	0.090	0.287	0.000	1.000
Whether trust in doctors for vaccination exists	14,956	0.702	0.457	0.000	1.000
Whether agreement with compulsory vaccination exists	14,933	0.468	0.499	0.000	1.000
Whether the government should bear all the cost for COVID–19 vaccination	14,813	0.843	0.364	0.000	1.000
Working status	14,966	0.727	0.445	0.000	1.000
Own income: Less than JPY 0.5 million	14,952	0.167	0.373	0.000	1.000
Own income: JPY0.5–0.99 million	14,952	0.074	0.262	0.000	1.000
Own income: JPY1–1.49 million	14,952	0.075	0.263	0.000	1.000
Own income: JPY1.5–1.99 million	14,952	0.056	0.231	0.000	1.000
Own income: JPY2–2.49 million	14,952	0.077	0.267	0.000	1.000
Own income: JPY2.5–2.99 million	14,952	0.063	0.243	0.000	1.000
Own income: JPY3–3.99 million	14,952	0.119	0.323	0.000	1.000
Own income: JPY4–4.99 million	14,952	0.109	0.312	0.000	1.000
Own income: Higher than JPY5 million	14,952	0.260	0.438	0.000	1.000
Household income: Less than JPY0.5 million	14,980	0.028	0.166	0.000	1.000
Household income: JPY0.5–0.99 million	14,980	0.012	0.109	0.000	1.000
Household income: JPY1–1.49 million	14,980	0.022	0.145	0.000	1.000
Household income: JPY1.5–1.99 million	14,980	0.031	0.173	0.000	1.000
Household income: JPY2–2.49 million	14,980	0.050	0.218	0.000	1.000
Household income: JPY2.5–2.99 million	14,980	0.051	0.219	0.000	1.000
Household income: JPY3–3.99 million	14,980	0.116	0.320	0.000	1.000
Household income: JPY4–4.99 million	14,980	0.122	0.328	0.000	1.000
Household income: JPY5–5.99 million	14,980	0.120	0.325	0.000	1.000
Household income: JPY6–6.99 million	14,980	0.092	0.289	0.000	1.000
Household income: JPY7–7.99 million	14,980	0.087	0.281	0.000	1.000
Household income: JPY8–8.99 million	14,980	0.069	0.254	0.000	1.000
Household income: JPY9–9.99 million	14,980	0.053	0.224	0.000	1.000
Household income: Higher than JPY10 million	14,980	0.148	0.355	0.000	1.000
Education: Junior high school	14,972	0.014	0.118	0.000	1.000
Education: High school	14,972	0.228	0.419	0.000	1.000
Education: Vocational college	14,972	0.124	0.330	0.000	1.000
Education: 2-year college	14,972	0.091	0.288	0.000	1.000
Education: Technical college	14,972	0.012	0.111	0.000	1.000
Education: 4-year college	14,972	0.471	0.499	0.000	1.000
Education: Graduate school	14,972	0.059	0.236	0.000	1.000
Approval of the Liberal Democratic Party	14,970	0.229	0.420	0.000	1.000
Degree of dissatisfaction with current politics	14,975	3.836	1.057	1.000	5.000
Subjective degree of right-leaning	14,634	6.183	1.396	1.000	10.000
Subjective social status	14,764	3.487	1.831	0.000	9.000

Gender has a value of 1 if the respondent is female and 0 otherwise. Working and marital status have a value of 1 if the respondent works and is married, respectively. The maximum value of the number of children is 5 such that an answer of “5” might include more than 5 children. Previous COVID-19 infection is also a dummy variable such that the mean 0.022 indicates that the mean probability of reporting a previous COVID-19 infection was 2.2%. Note that NA indicates how many respondents chose “I do not want to answer.” Previous experience with refusing and postponing vaccination are among the measures of general vaccination hesitancy (Larson et al. (2015)). The education and income strata are represented by dummy variables such that the mean values indicate the proportion of the sample in the stratum. The Liberal Democratic Party has been the ruling party for most of the period since its creation in 1955. The approval rates of the other parties, including the center-left Constitutional Democratic Party, the center-right government coalition Clean Government Party, and the leftist Japanese Communist Party, are less than 0.1 (10%). The values of dissatisfaction with current politics range between 5 (substantially dissatisfied) and 1 (substantially satisfied). When a respondent agreed with neither idea, we assigned a value of 3/5. The values of the subjective degree of individualism range between 1 (national interest is more prioritized than individual interest) and 5 (individual interest is more prioritized than national interest). The values of the subjective degree of right-leaning range between 10 (the rightest) and 0 (the leftest). The values of the subjective social status range between 10 (the highest) and 0 (the lowest). The difference between the sample number and 15,000 indicates the number of respondents who skipped the question.

For a comparison, we show the results of household income obtained by a survey by the Ministry of Health, Labour and Welfare of the government of Japan on the livelihood of 10,000 respondents, i.e., the “National Livelihood Survey” in 2019 in Table 3.^3^

**Table 3:** Distribution of household income in the National Livelihood Survey.

Income level	Number	Share
Total	10,000	100%
Less than JPY0.5 million	120	1.20%
JPY0.5–1 million	519	5.19%
JPY1–1.5 million	631	6.31%
JPY1.5–2 million	632	6.32%
JPY2–2.5 million	689	6.89%
JPY2.5–3 million	666	6.66%
JPY3–3.5 million	711	7.11%
JPY3.5–4 million	574	5.74%
JPY4–4.5 million	555	5.55%
JPY4.5–5 million	491	4.91%
JPY5–5.5 million	488	4.88%
JPY5.5–6 million	380	3.80%
JPY6–6.5 million	463	4.63%
JPY6.5–7 million	344	3.44%
JPY7–7.5 million	329	3.29%
JPY7.5–8 million	288	2.88%
JPY8–8.5 million	260	2.60%
JPY8.5–9 million	232	2.32%
JPY9–9.5 million	216	2.16%
JPY9.5–10 million	185	1.85%
Higher than JPY10 million	1,225	12.25%

*Source*: National Livelihood Survey 2019 by the Ministry of Health, Labour and Welfare of the government of Japan (https://www.e-stat.go.jp/stat-search/file-download?statInfId=000031957851&fileKind=1 Last accessed on July 5, 2021).

While our sample has a slightly denser distribution of the high-income level (higher than JPY10 million), the overall distribution of our survey does not greatly differ from that of the MHLW survey.

## 3 Estimation strategy

### 3.1 Identification

Let $Y_{ij}\left(a,d\right)$ be a potential outcome indicator, which takes = 1 if respondent *i* chooses alternative *j* and = 0 otherwise. $a=\left[a_{j},a_{-j}\right]$ is a set of two vectors of attributes, where ***a***_*j*_ and ***a***_−*j*_ are attribute vectors of alternative vaccines *j* and − *j* shown to respondent *i*, respectively. *d* is an indicator of the scenario, which takes = 1 if the scenario shown to the respondent assumed that the government was to make vaccination compulsory and = 0 if the scenario asked the respondent to choose a hypothetical vaccine for herself/himself.

Let ***A***_*ij*_ be a hypothetical vaccine selected by respondent *i*, and let *D*_*i*_ be a scenario shown to respondent *i*. Because observed ***A***_*ij*_ and *D*_*i*_ are randomized, the average marginal potential outcome is identified as follows:

(1) $$E\left[Y_{i}\left(a,d\right)\right]=E\left[Y_{ij}^{obs}|A_{ij}=a,D_{i}=d\right].$$

Regarding intervention of whether to make vaccination mandatory, our estimate of interest is obtained as follows:

(2) $$E\left[Y_{i}\left(a,d=1\right)-Y_{i}\left(a,d=0\right)\right].$$

In our estimation, we focus on the marginal value given a level of attribute *l* as follows:

(3) $$\underset{a_{-lj},a_{-j}}{\sum }E\left[Y_{i}\left(a_{lj},a_{-lj},a_{-j},d\right)\right]\times f\left(a_{-lj},a_{-j}\right),$$

where ***a***_−*lj*_ denotes a vector created by removing element *l* from ***a***_*j*_, and *f* denotes the joint density function.

### 3.2 Estimation

To estimate conditional means $E\left[Y_{ij}^{obs}|A_{ij}=a,D_{i}=d\right]$ in equation (1), the augmented inverse propensity score weight is employed. The conditional mean is defined as follows:

(4) $$E\left[Y_{ij}^{obs}|A_{ij}=a,D_{i}=d\right]=E\left[M_{ij}|A_{ij}=a,D_{i}=d\right],$$

where

$$M_{ij}=\mu_{d}\left(A_{ij}\right)+\frac{I\left(D_{i}\right)\times \left(Y_{i}^{obs}-\mu_{d}\left(A_{ij}\right)\right)}{e\left(A_{ij}\right)},$$

$\mu_{d}\left(a\right)=E\left[Y_{ij}^{obs}|A_{ij}=a,D_{i}=d\right]$, and $e\left(a\right)=E\left[D_{i}|A_{ij}=a\right]$. Thus we follow the estimation process such that:

1. $\mu_{d}\left(a\right)$ may essentially be estimated by any high-quality estimation method. In this case, 20-fold cross-fitted highly adaptive LASSO (Tibshirani (1996) and Benkeser and van der Laan (2016)) is employed. $e\left(a\right)$ is fixed as 0.5 in our experimental design since we did not allow the respondents to opt out when choosing between two hypothetical vaccines. The highly adaptive LASSO is implemented according to hal2009 (Hejazi et al. (2020)) with the default hyperparameter values.
2. We calculate the estimated score function ${\tilde{M}}_{ij}\left(d\right)={{\tilde{\mu }}_{d}}^{-k\left(i\right)}+\frac{I\left(D_{i}=d\right)\times \left(Y_{i}^{obs}-{{\tilde{\mu }}_{d}}^{\left(-k\left(i\right)\right)}\right)}{0.5}$, where ${{\tilde{\mu }}_{d}}^{-k\left(i\right)}$ is the average potential outcome, which is estimated by excluding the fold that includes respondent *i*.
3. We regress ${\tilde{M}}_{ij}$(d) on ***A***_*ij*_ to estimate $\sum_{a_{-j}}E\left[Y_{ij}\left(a_{j},a_{-j},\;d\right)\right]\times f\left(a_{-j}\right)$.

## 4 Results

### 4.1 Average marginal potential outcome

**Fig. 1:**
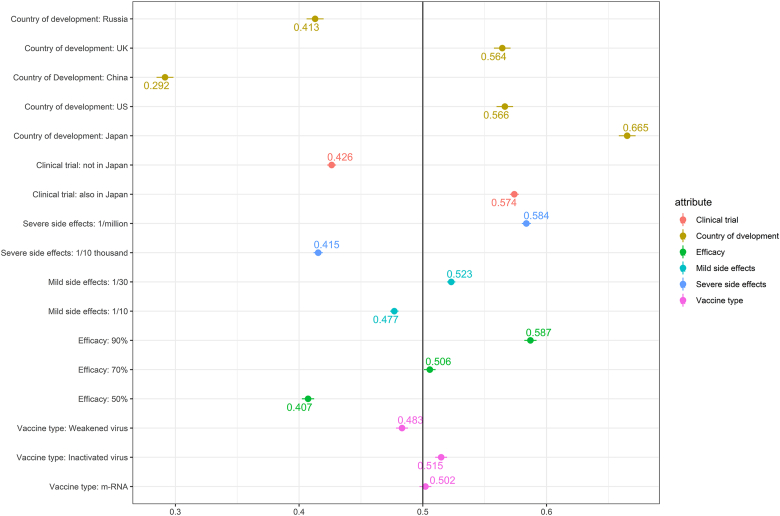
Average marginal potential outcomes of the vaccine attributes. *Notes*: Confidence interval is 95%.

The average marginal potential outcome, which is defined by equation (3), is presented in Fig. 1, which reports the results of the scenario of self-choice without considering a government ordinance to make vaccination compulsory.

The country of development is the factor most strongly associated with a preference for a vaccine. A change from a China-developed vaccine to a Russia-developed increased the respondents’ preference by 12.1 percentage points (from 0.292 to 0.413), a change to a UK-developed vaccine increased the choice probability by 27.2 percentage points (to 0.564), a change to a US-developed vaccine increased the choice probability by 27.4 percentage points (to 0.566), and a change to a Japan-developed vaccine increased the choice probability by 37.3 percentage points (to 0.665). In our setting, the reference point is 0.5 such that while both China-develpped and Russia-developed vaccines were penalized, the penalty was much greater for the China-developed vaccine. A similar penalty on China-developed vaccines has been reported in the US (Kreps et al. (2020) and Kreps and Kriner (2021)) and France (Schwarzinger et al. (2021)). Similar to the finding of Kreps et al. (2020) and Kreps and Kriner (2021) of a geopolitical penalty on China-developed vaccines, Japanese respondents penalized China the most, followed by Russia.

The second largest impacts were the location of the clinical trials and risk of severe adverse side effects. The preference for a hypothetical vaccine increased by 14.8 percentage points (from 0.426 to 0.574) if the clinical trials were conducted in Japan than in other countries, and the probability of preferring a vaccine increased by 16.9 percentage points (from 0.415 to 0.584) if the probability of severe adverse side effects decreased from 1 per ten thousand to 1 per 1 million.

Third, efficacy had modest impacts. An increase in efficacy in preventing severe symptoms, such as hospitalization or death, from 50% to 70% raised the choice probability by 9.9 percentage points (from 0.407 to 0.506), and an efficacy of 90% raised the choice probability by 18.0 percentage points (to 0.587).

Fourth, the vaccine type did not affect the choice probability of a vaccine. Our randomized conjoint design includes both efficacy and side effect risk. The respondents chose a hypothetical vaccine between two vaccines given the efficacy and side effect risk specified by hypothetical vaccine attributes. Hence, the two following possibilities could explain the irrelevance of the vaccine types we observed.

1. The respondents’ concerns regarding the probabilistic factors of vaccine types all depend on the efficacy and side effect risk. Therefore, our conjoint design including efficacy and side effect risk as attributes resolves all the risk relevant to the respondents such that the vaccine types conditional on the specified efficacy and side effects were irrelevant to the respondents.
2. While the respondents were subjectively concerned about probabilistic factors other than the specified efficacy and side effect risk, they did not have enough knowledge to differentiate the vaccine types such that the vaccine types were not associated with the probability of a hypothetical vaccine being chosen.

We cannot identify which of the two possibilities is more likely by our design.

As a robustness check, we estimated the average marginal component effects among the respondents aged 60 or older, with high school or less as the highest degree of education, and residing outside of metropolitan regions (Greater Tokyo: Tokyo, Saitama, Chiba, and Kanagawa prefectures; Greater Nagoya: Gifu, Aichi, and Mie prefectures; and Greater Osaka: Kyoto, Osaka, Gyogo, and Nara prefectures), as shown in the appendix. The findings were qualitatively the same as those of the entire sample.

### 4.2 Treatment effects

**Fig. 2:**
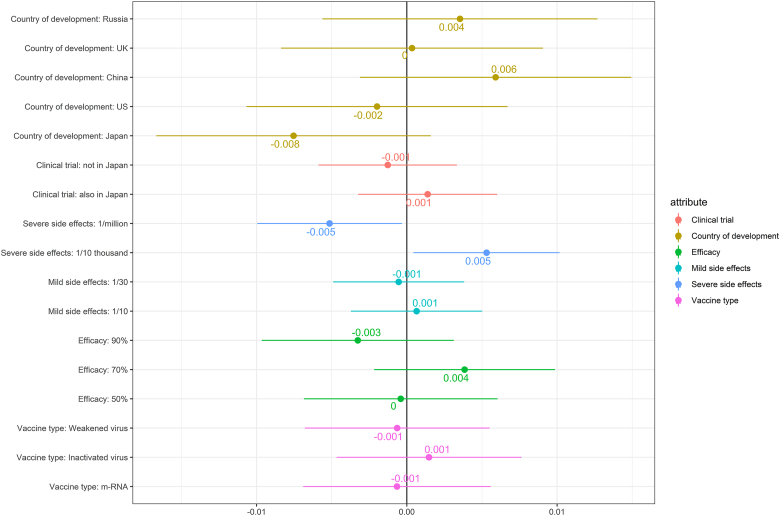
Differences in the average marginal component outcomes of the vaccine attributes. *Notes*: Confidence interval is 95%.

Finally, we estimate equation (2) by equation (3) such that

(5) $$\underset{a_{-lj},a_{-j}}{\sum }E\left[Y_{i}\left(a_{lj},a_{-lj},a_{-j},1\right)-Y_{i}\left(a_{lj},a_{-lj},a_{-j},0\right)\right]\times f\left(a_{-lj},a_{-j}\right)$$

by regressing ${\tilde{M}}_{ij}\left(1\right)-{\tilde{M}}_{ij}\left(0\right)$ on ***A***_*ij*_. The results are presented in Fig. 2.

As indicated in equation (5), Fig. 2 shows the difference in choosing a hypothetical vaccine between the cases when it was optional and when it was government mandated by subtracting the former’s value from the latter’s value. While the effects of making the vaccine compulsory are generally modest, the penalty on a China-developed vaccine decreased by 0.6 percentage points, the penalty on a Russia-developed vaccine decreased by 0.4 percentage points, and the penalty on a high-risk vaccine (severe adverse side effects in 1 per 10 thousand) decreased by 0.5 percentage points. Additionally, the support for a hypothetical vaccine with modest efficacy (70% efficacy against severe symptoms) increased by 0.4 percentage points.

Fig. 2 shows the difference between optional vaccination and mandatory vaccination. Thus, negative or slight effects do not indicate negative or irrelevant support for a vaccine. Instead, an estimated value indicates whether the preference for a vaccine with the attribute increases if vaccination was mandated. For instance, a rise in support for a hypothetical vaccine with 70% efficacy does not indicate that the vaccine was preferred to the other vaccine but that it would have been more preferred if vaccination was mandated than otherwise. The potential margins of the increase due to compulsory vaccination are larger for disliked vaccines. In summary, a rise in preference due to mandatory vaccination captures a rise in preference because it is impossible to avoid less preferred vaccines; thus, more people accept the vaccine. One possible interpretation is that people tend to accept less effective or riskier vaccines if vaccination is mandatory because mandatory vaccination eliminates free-riding concerns when building up herd immunity.

**Fig. 3:**
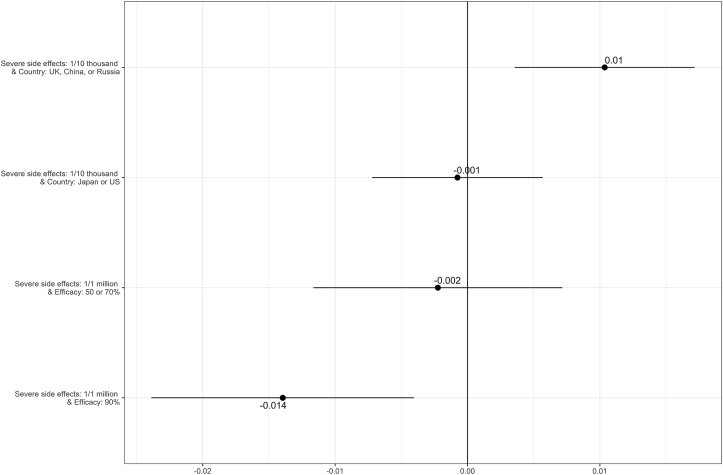
Impacts of compulsory vaccination on interactions among vaccine attributes. *Notes*: Confidence interval is 95%.

Fig. 3 reports the impact of making a vaccine compulsory on the interactions among the four critical elements that affected preference the most. The reported interaction is detected by a recursive partitioning with a depth equal to 2 in which the objective function is the root mean squared error (RMSE) of the fitted values and $\left({{\tilde{\mu }}_{1}}^{-k\left(i\right)}-{{\tilde{\mu }}_{0}}^{-k\left(i\right)}\right)$.

The results indicate that making vaccination compulsory increases the choice probability of China-, Russia-, and UK-developed vaccines with severe adverse side effects of 1 per 10 thousand by 1 percentage point but does not affect Japan- or US-developed vaccines, vaccines whose severe adverse side effects of 1 per 1 million, and the modestly effective vaccines whose efficacy against severe symptoms is 50 or 70%. The rise in support for riskier and China, Russia, UK-developed vaccines is primarily explained by a decrease in the support for vaccines whose probability of severe adverse side effects is 1 per 1 million and whose efficacy against severe symptoms is 90%.

We screened the interactions between the vaccine attributes and background characteristics, including previous COVID-19 infection experience, general attitudes toward vaccination, past vaccination or vaccination refusal experience, trust in doctors’ advice, trust in the government’s vaccine licensing, educational background, income, and political positions, as indicated in Table 2, but these characteristics did not have substantial impacts on the preference for hypothetical vaccines. The irrelevance of past vaccination experience is consistent with the results reported by Kreps and Kriner (2021). Therefore, we conclude that the attributes of vaccines we discussed above impact vaccination preferences across diverse background characteristics, including general vaccine hesitancy.

## 5 Conclusions

As noted by Matta and Nov (2020), better scientific communication between the public and authorities is effective as a better approach to containing the pandemic. Better communication could be achieved by either enhancing public knowledge or deepening the authorities’ understanding of the public’s fear. Regarding this issue, Niño et al. (2021) and Saied et al. (2021) specified the factors that could render people hesitant about vaccination. If the factors are within the authorities’ discretion, they could better address the issue. We attempted to provide such information to relevant policy makers.

Domestic development and domestic clinical trials are strongly preferred by Japanese adults. However, such vaccines cannot be expected in the very near future. The US-developed vaccines are preferred to other vaccines of foreign origin. Regarding compulsory vaccination, riskier, modestly effective, China-developed, Russia-developed, and UK-developed vaccines gain increased support. Suppose Japan cannot obtain a sufficient quantity of US-developed vaccines and must deploy riskier, modestly effective, China-developed, Russia-developed, or UK-developed vaccines as substitutes at the transitional stage. In that case, compulsory vaccination could be an effective way to reduce free-riding concerns to achieve herd immunity. If the government should deploy less preferred vaccines, then compulsory vaccination might help.

The channel of externality, which reflects positive spillover effects such that people might seek to free ride on others’ vaccination, was asymmetric. If the reduction in the externality concerns channel was symmetric to both removing negative externality (free-riding concerns) and materializing positive externality (“win-win” scenario between vaccine supporters and the vaccine hesitant), then legislation for compulsory vaccination would have increased support for the most effective vaccines (90% efficacy against severe symptoms), which was not observed in our experiment. These findings suggest that the public primarily expects the authorities to curb free-riding. Regarding the vaccine hesitant who might want to be protected by herd immunity but want to avoid vaccination, the public wants the government to force the vaccine hesitant to get vaccinated.

The primary limitations of our research are related to the irrelevance of possible learning through vaccination experience and irrelevance of the vaccine types. As presented in Kreps and Kriner (2021), past vaccination experiences were not associated with the choice of hypothetical vaccines in our experiment. While we cannot identify the possible reasons, this finding might indicate that vaccine preference does not necessarily evolve over vaccination experience. If so, authorities should not wait to provide appropriate vaccines until the public learns. Although active instructions and suggestions seem to be necessary, the issue is beyond our design, and inquiry regarding this issue is left for future research.

Additionally, while we did not observe an association between the vaccine types and the likelihood of choice, we cannot identify whether this finding was because the respondents’ were only concerned about efficacy and side effect risk specified in our design, rendering the vaccine type irrelevant, or because the respondents did not have enough knowledge to differentiate the vaccines even though they cared about factors other than efficacy and side effect risk specified in our design. Knowing which explanation is correct could be helpful for the authorities to convey appropriate vaccines. However, this issue is left for future research.

## Author statement

Kawata and Nakabayashi set the aim of the survey.

Kawata and Nakbayashi designed the randomized conjoint experiment and background characteristics survey.

Kawata and Nakabayashi implemented the survey experiment designed by Kawata and Nakabayashi.

Kawata analyzed the collected data and provided the primary results in an experimental report.

Nakabayashi extended Kawata's experimental report to a draft of manuscript.

Kawata and Nakabayashi discussed possibility of additional analyses and added them.

Kawata and Nakabayashi finalized the manuscript.

## Conflicts of interest and ethical review

• The authors declare that they have no relevant or material financial interests that relate to the research described in this paper.
• The Ethical Review Board of the Institute of Social Science, The University of Tokyo approved this study.

## Figures and Tables

**Figure A1:**
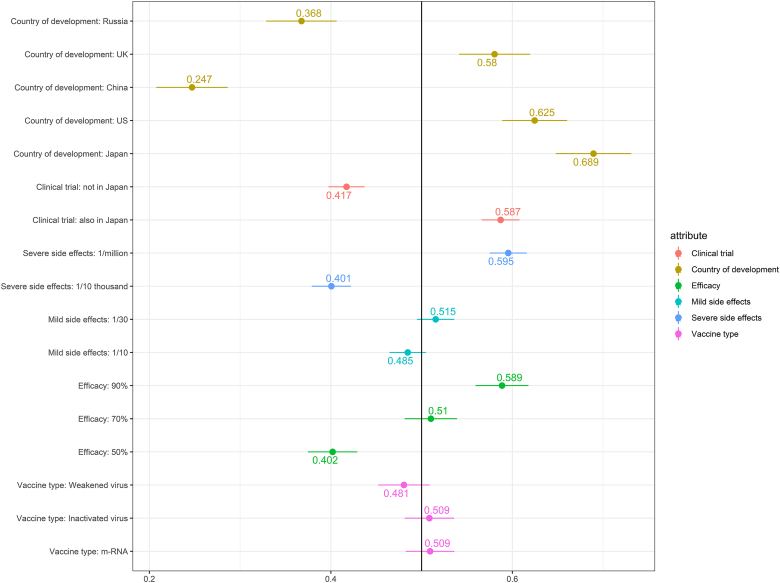
Average marginal potential outcomes of the following vaccine attributes: less educated, less urbanized, and older samples. *Notes*: Confidence interval is 95%.

## Appendix

We conducted this survey online. As additional concerns, educational background, Internet literacy that might depend on age, and urbanization might have affected the results. Thus, we calculated the main result (Fig. 1) in a restricted sample such that the respondents’ highest degree was high school or less, the respondents’ residence was outside of metropolitan regions, i.e., greater Tokyo (Tokyo, Saitama, Chiba, and Kanagawa prefectures), greater Nagoya (Gifu, Aichi, and Mie prefectures), and greater Osaka (Kyoto, Osaka, Nara, and Hyogo prefectures), and the respondents were aged 60 or older. As shown in Fig. A1, China-developed vaccines are less preferred to Japan-developed vaccines by 44.2 percentage points (from 0.689 to 0.247), and Russia-developed vaccines are less preferred by 32.1 percentage points (from 0.689 to 0.368). Clinical trials conducted in Japan increased the vaccine preference more by 17.0 percentage points (from 0.417 to 0.587). Severe adverse side effects in 1 per million increased the preference for the vaccine more than severe adverse side effects in 1 per 10 thousand by 19.4 percentage points (from 0.401 to 0.595). An efficacy increase from 50% to 90% increased the preference by 18.7 percentage points (from 0.402 to 0.589). The vaccine types did not show statistically significant differences. The results are qualitatively consistent with those shown in Fig. 1.

## Footnotes

1 https://insight.rakuten.co.jp/en/Last accessed on July 27, 2021.

2 https://insight.rakuten.co.jp/download/PanelProfile_EN.pdf and https://insight.rakuten.co.jp/download/PanelCharacteristicSurveyEN.pdf Last accessed on July 27, 2021.

3 Description of the survey: https://www.mhlw.go.jp/toukei/list/20-21.html Last accessed on July 5, 2021.
